# Identification of anti-inflammatory fractions of *Geranium wilfordii* using tumor necrosis factor-alpha as a drug target on Herbochip® – an array-based high throughput screening platform

**DOI:** 10.1186/s12906-015-0665-9

**Published:** 2015-05-12

**Authors:** Min Huang, Pei-Wun Yao, Margaret Dah-Tysr Chang, Sim-Kun Ng, Chien-Hui Yu, Yun-feng Zhang, Meng-Liang Wen, Xiao-yuan Yang, Yiu-Kay Lai

**Affiliations:** Yunnan Baiyao-Herbcopoeia Laboratory Inc, 51 Xi-Ba Road, Kunming, Yunnan China; Yunnan Institute of Microbiology, Yunnan University, Kunming, China; Institute of Biotechnology and Department of Life Science, National Tsing Hua University, Hsinchu, Taiwan; Institute of Molecular and Cellular Biology and Department of Medical Science, National Tsing Hua University, Hsinchu, Taiwan; School of Life Sciences, Yunnan Normal University, Kunming, Yunnan China

**Keywords:** Geraniaceae, *Geranium wilfordii*, Anti-inflammatory, Anti-rheumatoid arthritis, Herbochip, TNF-alpha

## Abstract

**Background:**

*Geranium wilfordii* is one of the major species used as Herba Geranii (lao-guan-cao) in China, it is commonly used solely or in polyherbal formulations for treatment of joint pain resulted from rheumatoid arthritis (RA) and gout. This herb is used to validate a target-based drug screening platform called Herbochip® and evaluate anti-inflammatory effects of *Geranium wilfordii* ethanolic extract (GWE) using tumor necrosis factor-alpha (TNF-α) as a drug target together with subsequent *in vitro* and *in vivo* assays.

**Methods:**

A microarray-based drug screening platform was constructed by arraying HPLC fractions of herbal extracts onto a surface-activated polystyrene slide (Herbochip®). Using TNF-α as a molecular probe, fractions of 82 selected herbal extracts, including GWE, were then screened to identify plant extracts containing TNF-α-binding agents. Cytotoxicity of GWE and modulatory effects of GWE on TNF-α expression were evaluated by cell-based assays using TNF-α sensitive murine fibrosarcoma L929 cells as an *in vitro* model.

**Results:**

The *in vivo* anti-inflammatory effects of GWE were further assessed by animal models including carrageenan-induced hind paw edema in rats and xylene-induced ear edema in mice, in comparison with aspirin. The hybridization data obtained by Herbochip® analysis showed unambiguous signals which confirmed TNF-α binding activity in 46 herbal extracts including GWE. In L929 cells GWE showed significant inhibitory effect on TNF-α expression with negligible cytotoxicity. GWE also significantly inhibited formation of carrageenan-induced hind paw edema and xylene-induced ear edema in animal models, indicating that it indeed possessed anti-inflammatory activity.

**Conclusion:**

We have thus validated effectiveness of the Herbochip® drug screening platform using TNF-α as a molecular target. Subsequent experiments on GWE lead us to conclude that the anti-RA activity of GWE can be attributed to inhibitory effect of GWE on the key inflammatory factor, TNF-α. Our results contribute towards validation of the traditional use of GWE in the treatment of RA and other inflammatory joint disorders.

## Background

The dried aerial parts of *Geranium wilfordii* Maxim (GW), *Erodium stephanianum* Willd., and other congenic plants including *G. Carolinianum* in the Geraniaceae family are used as Chinese medicinal plant lao-guan-cao or *Herba geranii/Herba erodii*. According to Pharmacopoeia of People’s Republic of China 2010 [1], GW is acrid, bitter, neutral; and liver, kidney, spleen meridian entered. GW is effective for eliminating wind-dampness, unblock meridians, as well as stopping diarrhea and dysentery, thus it is clinically used for treatments of rheumatoid arthritis (RA), spasm numbness, myalgia, *etc*., ailments that related to inflammation, swelling, pain, and other discomforts in muscles, bones, and joints. GW can be used in polyherbal formulation such as in Yunnan Baiyao or alone such as in the lao-guan-cao paste/cream (*Unguentum Geranii*) for topical therapy of the aforementioned ailments, particularly RA [1]. More recent pharmacological studies indicate that this herb, together with its congenic plants, also possesses anti-oxidative, anti-viral, anti-cancer, antinociceptive and others effect [2,3]. Unfortunately, despite of its wide application in traditional Chinese medicine, the molecular mechanism underlying its efficacy has seldom been reported.

RA is a progressive joint damaging and disabling disease characterized by pain, swelling, and stiffness of the synovial joints. This autoimmune disease is resulted from dysregulations of both innate and adaptative immunity, *i.e.*, interactions between genetic and environmental factors are involved [4-6]. Cumulating evidences indicate that RA is also a chronic inflammatory disease involving many cytokines that act both in series and in parallel, a phenomenon known as cytokine network redundancy [4]. Among the cytokines involved tumor necrosis factor-alpha (TNF-α) plays a pivotal role in the pathogenesis and treatments of RA [7,8]. TNF-α inhibitory biologics disease-modifying anti-rheumatic drugs (DMARDs) such as infliximab, etanercept, adalimumab, certolizumab and golimumab produced significant therapeutic effects in large numbers of RA patients [7]. The successful clinical applications of these TNF-α inhibitors firmly demonstrate the fact that TNF-α is indeed the key inflammatory mediator in RA. Unfortunately, these immune-modulators will cause adverse effects including infusion reactions, injection site reactions and immunogenicity, as well as increased risks in malignance, cardiovascular, liver and other autoimmune diseases [9]. Before the use of these DMARDs, more conventional drugs used for the management of RA are steriods and non-steroidal anti-inflammatory drugs (NSAIDs); but their long-term use is well known to be associated with serious adverse effects as well. Thus, RA patients often seek alternative ways and means for symptomatic relief and are amongst the highest users of complementary and alternative medicines (CAMs) [10,11].

A decade ago, a WHO [12] report stated that more than 80% of the world market relies on herbal medicinal products. In fact, use of herbal medicines is an ancient practice in almost all cultures. In recent years use of herbal products either as dietary supplements, medical foods, or therapeutics, has become a common trend around the world or functional effects have been and continue to be rigorously studied [13]. Unfortunately, very few biological targets have been unanimously identified for the vast amounts of natural products studied. The most well-known drug target for a number of natural products may be α-tubulin in microtubules. Tubulin-binding phytochemicals such as vincristine, vinblastine, paclitaxel, and ixabepilone are developed to be frontline anti-cancer drugs [14]. Another highly publicized example is the identification of SERCA of *Plasmodium falciparum* as the drug target of artemisinins (qinghaosu) [15] from the antimalarial herb *Artemisia annua* L. (qinghao) [16,17]. It is worth to note that all of the target identifications, if any, for herbal compounds are likely resulted after functional identification and screening. In 2003, Chang *et al.* published a microarray-based drug screening technology (Herbochip®) which allowed functional characterization of herbal compounds fractionated by HPLC using a defined protein drug target in a reverse screening fashion. The prototype of this technology was reported to be used for finding anti-TNF-α active herbal fractions as mentioned in a patent application [17]. Subsequently, herbochips containing HPLC fractions from *Sophora flavescens* (SFE-herbochip) were screened using cytochrome P450 3A4 (CYP450 3A4) as a molecular target, hoping to obtain anti-CYP450 3A4 active components that might be useful in the design of regimens for the treatment of human immunodeficiency virus (HIV) infection [18]. Herein, we present a refined version of the Herbochip® platform and report successful identification of the binding activity toward TNF-α in 46 out of 82 selected herbal extracts. Furthermore, the anti-TNF-α and anti-inflammatory effects of the GWE were respectively attested by *in vitro* and *in vivo* assays. Our results present a platform that can be used for drug screening with known drug targets and contribute towards validation of the traditional use of GWE in the treatment of RA and other inflammatory joint disorders.

## Methods

### Cell line, chemicals and biochemicals

L929 (NCTC clone 929, derivative of Strain L) cells line was purchased from the China Center for Type Culture Collection (CCTCC, number: GDC034). Aspirin was from Shandong Xinhua Pharmaceutical Co. Ltd., Shangdong, China. Actinomycin D (ActD) was from Beyotime Institute of Biotechnology, Jiangsu, China. Sodium hydrogen carbonate, dimethyl sulfoxide, Tween 20, sodium chloride, and xylene were from Sinopharm Chemical Reagent Co. Ltd., Shanghai, China. Other reagents and chemicals were from Sigma-Aldrich, USA.

### Plants and plant extracts

For primary screenings, most of the plant materials of 82 selected Chinese herbs were either self-collected or purchased from market (Table 1). Specifically, the plant materials of *Geranium wilfordii* Maxim were obtained from Yannan Baiyao Group Tianzihong Pharmaceutical Co. Ltd. (Kunming, Yunnan, China) and identified by Yunnan Institute of Materia Medica (Kunming, Yunnan, China) according to The Pharmacopoeia Commission of PR China 2010 [1]. Voucher specimens of the whole plant of *G. wilfordii* as well as all plant materials screened were stored in our laboratory (Table 1). Plant extracts for herbochip screening were prepared as follows. For each preparation 50 g dried plant materials was ground to powder and extracted with 1 L 50% ethanol (1:20 w/v) at room temperature for three days. The extract was concentrated to 2.5% of its original volume by vacuum evaporation (R200, Buchi, Swiss Confederation) at approximately 60°C and then dried by lyophilization (LGJ-10D, Four-Ring Science Instrument Plant Beijing Co., Ltd., China). The dry powder was kept at 4°C until use. Since large amount of ethanolic extract of *G. wilfordii* (GWE) was needed in subsequent experiments, the starting material was increased to 3 kg for preparation of GWE according to the same protocol. The positive control, aspirin (95% pure), was obtained from the Shandong Xinhua Pharmaceutical Co., Ltd. (Zibo, Shandong, China).

**Table 1:** **Hybridization reactivity of herbal extracts using TNF-α as a probe on herbochips**

**Herbal extracts**	**Parts used**	**Specimen number**	Hybridization reactivity*
*Acacia spinosa*	stem	B0210A0402	-
*Acer garrettii*	root	A1108A0304	*+++*
*Achyranthes bidentata*	root	A0604A0082	-
*Aconitum carmichaeli*	root	A1104A0095	-
*Aconitum kusnezoffii*	root	A1203A0096	-
*Allemanda blanchetii*	leaf	C1108A0303	-
*Amomum kravanh*	pericarp	M0704A0085	+
*Angelica pubescens*	root	A0704A0083	-
*Angelica sinensis*	root	A0904A0080	-
*Atractylodes chinensis*	stem and root	A0604A0084	-
*Atractylodes macrocephala*	stem and root	A0804A0081	-
*Blinkworthia lycioides*	stem and leaf	H1009B0202	+
*Cachlosbermum regium*	flower	D0710C0629	++
*Callisia fragrans*	leaf	C1009C0574	-
*Campylotropis yunnanensis*	stem and leaf	H1209C0591	+++
*Centrolobium ochroxylum*	leaf	C1009C0578	+++
*Chaenomeles speciosa*	fruit	E0904C0131	++
*Chaetocarpus castanocarpus* var. *pubescens*	fruit	E0710C0634	-
*Cinchona succirubra*	stem	B0710C0637	-
*Cinnamomum burmannii*	stem	B0210C0621	+++
*Cinnamomum cassia*	stem	B0904C0130	++
*Citrus reticulata*	pericarp	M0404C0132	++
*Clematis chinesis*	root	A1104C0144	-
*Clinacanthus nutans*	stem and leaf	H1009C0569	-
*Cochlospermum vitifolium*	stem	B0210C0624	+++
*Codiaeum variegatum* cv. Sunny Star	leaf	C1209C0601	-
*Colubrina arborescens*	stem	B0210C0617	+
*Curcumorpha longifolia*	whole plant	G1108C0451	-
*Cyrtomium fortunei*	stem and root	A1003C0140	++
*Derris tonkinensis*	stem	B1009D0207	+/−
*Diospyros xishuangbannaensis*	stem	B0710D0237	+++
*Dipsacus asperoides*	root	A0604D0048	-
*Eucommia ulmoides*	bark	J0604E0035	*+*
*Garcia nutans*	stem	B0609G0123	*+*
*Ephedra sinica*	stem	B0604E0036	++
*Garcinia bancans*	leaf	C1009G0153	*+++*
*Gardenia erythroslada*	stem	B0609G0125	*+*
*Gastrodia elata*	root	A1104G0038	-
*Gentiana crassicaulis*	root	A1004G0037	-
*Geranium wilfordii*	whole plant	G0204E0037	++
*Gnetum gnemon* var. *tenerum*	fruit	E1108G0099	+/−
*Harpullia arborea*	stem	B0210H0165	-
*Hibiscus rosa-chinensis*	stem	B1009H0157	-
*Homalomena occulta*	stem and root	A0604H0034	++
*Illicium difengpi*	velamen	L1103I0012	+++
*Ixora duffii* cv. Super King	stem	B1009I0065	+++
*Lantana camara* var. *flava* Moldenke	stem and leaf	H1108L0127	-
*Leea hispida*	stem	B1009L0163	+++
*Litsea lancifolia*	leaf	C1108L0138	+++
*Lysimachia garrettii*	whole plant	G0609L0142	++
*Millettia chenkangensis*	fruit	E1209M0293	+
*Millettia pulchra*	bark	J1108M0229	+
*Mussaenda flava*	stem and leaf	H1009M0281	+++
*Paris polyphylla* Smith var. *yunnanensis*	root	A0610P0053	-
*Phellodendron amurense*	bark	J0704P0079	-
*Pittosporum formosanum hayata* var. *hainanensis gagnep*	stem	B0210P0403	+
*Pogostemon cablin*	underground	G0304P0078	+/−
*Polygonum multiflorum*	root	A0304P0089	++
*Poria cocos*	sporocarp	G0904P0084	-
*Pueraria thomsonii*	root	A0904P0080	-
*Pygeum latifolium*	stem	B0710P0411	+++
*Randia wallichii*	stem	B1209R0125	+
*Rehmannia glutinosa*	root	A1104R0026	-
*Rheum palmatum*	root	A0804R0023	+++
*Rivina humilis* var. *fructu*	stem/leaf	H1108R0087	+/−
*Saraca thaipingensis*	stem	B0210S0338	+++
*Schefflera menglaensis*	stem	B0210S0326	+
*Seseli mairei*	root	A0604S0069	-
*Shirakiopsis indica*	fruit	E0210S0337	-
*Sinomenium acutum*	stem	B1103S0064	+
*Syzygium caryophyllaceum*	stem	B1009S0300	++
*Taraxacum officnala*	whole plant	G1208T0157	-
*Taxillus chinensis*	stem	B0904T0042	+++
*Ternstroemia gymnanthera* var. *wightii*	bark	J0308T0129	+++
*Tricalysia mollissima*	root	A1108T0155	++
*Typhonium giganteum*	root stem	B1003T0043	-
*Uvaria cordata*	stem	B1009U0033	+++
*Ventilago madraspatana*	stem	B1108V0050	+++
*Vitex glabrata*	fruit	B1108V0047	+/−
*Vitex glabrata*	stem	E0210V0059	++
*Vitis balanseana*	root	A0409V0053	+++
*Wrightia fruticosa*	stem	B0210W0045	-

* “-” = negative; “+/-” = inconsistent results; “+” = weak positive, Fluorescence Value (FV) < 200; “++” = moderate positive, FV between 200 and 1000; “+++” = strong positive, FV >1000.

### Preparation of the herbochips and screening for TNF-α binding activity

Fractionation of the plant extract by HPLC: The plant extracts obtained above, including that made from *Geranium wilfordii* (GWE), were dissolved in 50% ethanol to a concentration of 50 mg/mL. The sample was then resolved into 96 fractions by a HPLC system equipped with a C18 column, an Agilent1100 instrument, a quaternary pump, a manual injector, a UV detector and a workstation for peak identification and integration (Agilent Technologies, USA). The column used was a Hypersil ODS C-18 analytical column (4.6 × 250 mm, 5 μm) and the detection wavelength was set at 254 nm. For each fractionation, 50 μL sample was loaded and the elution was carried out by a water to EtOH gradient at a constant flow rate of 0.75 mL/min at 30°C for 96 min as follows: 0-50% EtOH (90 min), 50-100% EtOH (6 min). One-minute fractions were collected and then 150 μL each fraction was transferred into a 96-well microplate (Corning Incorporated, USA). Thus each plate represented a complete HPLC run of the fractions/samples load. The plates were vacuum-dried by a Speed Vac (Savant, Thermo Fisher Scientific, Germany) and stored at 4°C until use.

**Figure 1:**
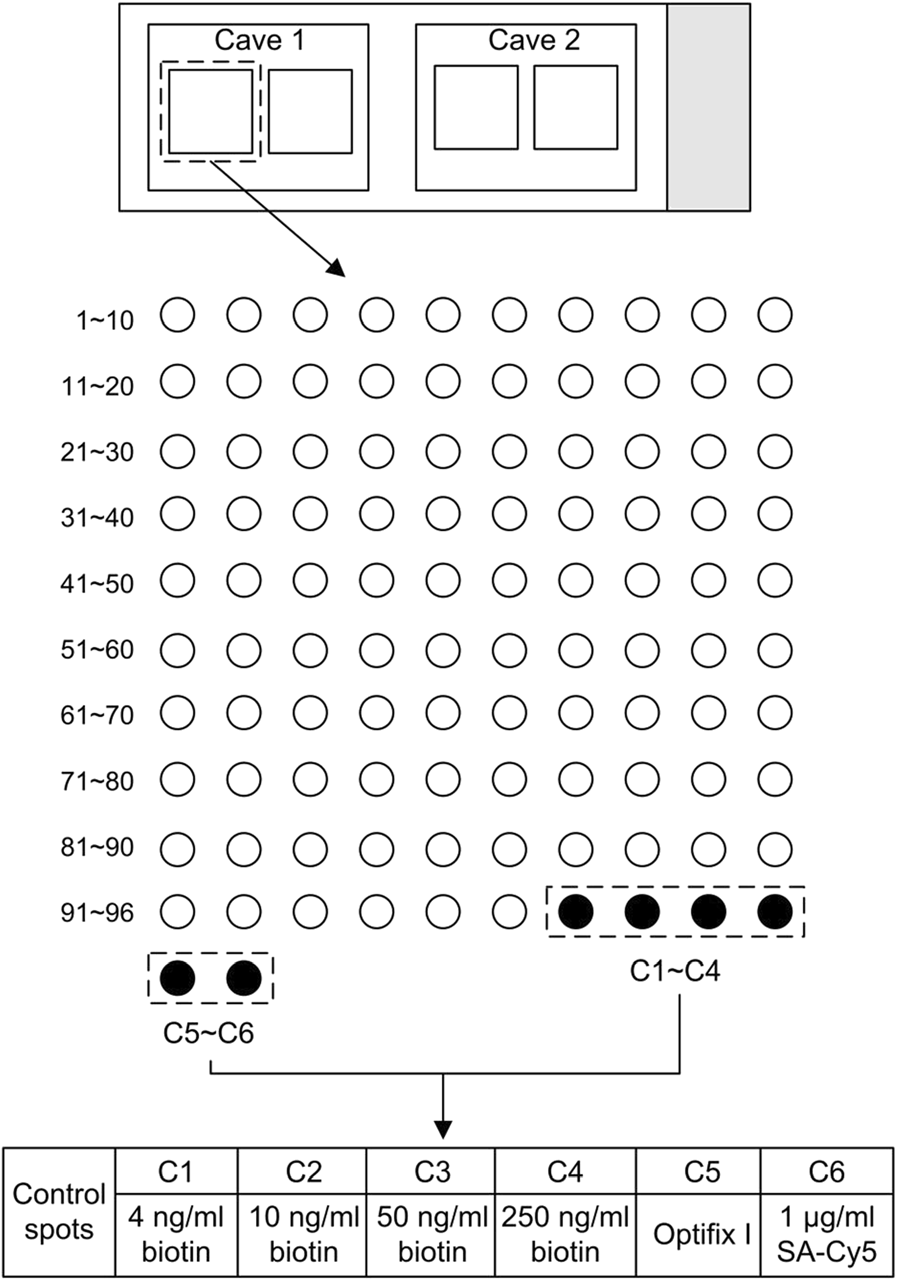
Schematic illustration of a herbochip. In each of the plastic slides (25 mm x 75 mm x 1.4 mm), two caves (1 and 2) were molded and used for control and tested samples. HPLC fractions were spotted into each cave in duplicates. The 96 fractions from HPLC were formatted as spots 1 – 96 and there were six control spots (C1 – C6) as illustrated.

Fabrication of herbochips - arraying the HPLC fractions on plastic slides: The blank chips were manufactured by mode injection followed by surface-activation [17]. Briefly, the molded plastic slides (25 mm × 75 mm × 1.4 mm) with two caves (Figure 1) were made of polystyrene and surface-activated by consecutive treatments with glutaraldehyde (0.4%, pH 5.0; room temperature, 4 h; washed in H_2_O), NH_4_OH (3 M, pH 11.0; 60°C, 4 h), and 1,4-butanediol diglycidyl ether (100 mM, pH 11.0; 37°C, overnight). After treatments, the slides were washed with 0.1 M NaHCO_3_ (pH 8.0), dried, sealed and stored at 4°C until use (blank chips). Prior to spotting, 30 μL OptiFix I (40 mM sodium borate, 0.1 M PBS, 0.01% Tween 20, 20% DMSO, pH 10.0) was added to each well of the 96-well plates. The samples were then spotted onto the blank chips using an automatic arrayer (Biodot A101, Shuai Ran Precision, Taiwan) according to the format shown in Figure 1. For controls, biotinamidocaproyl hydrazide (Sigma, USA) was serially diluted to 4, 10, 50, 250 ng/mL with OptiFix I (Figure 1, C1 to C4), OptiFix I alone (Figure 1, C5), 1 μg/mL Cy5-labeled streptavidin (SA-Cy5, GE Health) in OptiFix II (50 mM sodium tetraborate, 0.1 M PBS, 0.01% Tween 20, pH7.4) were also processed alongside with the above samples (Figure 1, C6). The slides were then air-dried and stored at 4°C overnight. After being blocked with blocking buffer (0.1 M ethanolamine, 0.1 M sodium tetraborate) for 1 h, they were washed four times with TBST (50 mM Tris · HCl, 0.15 M NaCl, 0.05% Tween 20, pH 7.5), rinsed with double distilled water four times, dried at 30 min and stored at 4°C until used. The resulted slides, with HPLC fractions arrayed, were designated as herbochips and named with the herbs used. For instance, GWE-herbochip is a herbochip that was arrayed with GWE.

Screening TNF-α binding activities in the plant extracts by using the Herbochip® platform: Purified TNF-α was purchased from PrimeGene Technical (Shanghai, China). For preparation of biotinylated TNF-α, 1 mg protein was dissolved in 1 mL coupling buffer (100 mM NaHCO_3_, pH 8.0), and 50 μL the BNHS solution (2 mg/mL biotinamidohexanoic acid *N*-hydroxysuccinimide ester in DMSO) was added. The biotinylation reaction was carried out for 1 h on ice and then stopped by adding equal volume of glycine (50 mM in coupling buffer). The mixture was then dialyzed against coupling buffer to remove unreacted BNHS and excess glycine. For hybridization, each cave on the chips was covered by a cover glass (22 × 22 mm) under which 20 μL biotinylated-TNF-α in TBST (50 mM Tris–HCl, pH 7.5; 0.15 M NaCl, 0.05% Tween 20) or TBST alone was added. The slides were then incubated at 37°C for 1 h. While placed in a slide washing jar, the slides were washed in TBST (4 × 2 min), rinsed in water (4 × 2 min) on a horizontal shaker at 80 rpm at room temperature, and then dried at 37°C (30 min). The same hybridization procedure was used in the subsequent reaction with Cy5-labeled streptavidin (SA-Cy5) where 20 μL 2.5 μg/mL SA-Cy5 in TBST was used. The dried slides were scanned by a laser scanner (GenePix4100 A, Axon, USA) and the fluorescent intensity of red spots in the image was analyzed at emission wavelength of 635 nm by GenePix 5.0 Software. The fluorescence value (FV) of each hybridization reaction was then recorded.

### Cells and cell treatments – cytotoxicity and anti-TNF-α activity of GWE

The TNF-α sensitive murine fibrosarcoma L929 cells were used for determination of cytotoxicity and inhibitory effect on TNF-α of the samples. L929 cells were cultured in RPMI-1640 medium (Gibco, Grand Island, NY, USA) supplemented with 10% fetal bovine serum (FBS), 100 μg/mL streptomycin, 100 U/mL penicillin, and 0.03% L-glutamine, and maintained at 37°C in humidified air containing 5% CO_2_. All the experiments were performed on logarithmically growing cells. For treatments, the L929 cells were seeded into 96-well microtiter plates at a concentration of 1.5 × 10^4^ cells/well in 100 μL of culture media and allowed to recover for 24 h. The cells were pretreated with ActD at 2 μg/mL for 1 h and then with four twofold serial dilutions of the test drug (GWE dissolved in culture media) for an additional 24, 48, and 72 h. Cell viability was determined by incubation with 3-(4,5-dimethylthiazol-2-yl)-2,5-diphenyl tetrazolium bromide (MTT) (0.5 mg/mL) for 1 h, solubilization in dimethyl sulfoxide (DMSO), and spectrophotometric measurement at 550 nm [19]. The cytotoxicites of the test drugs were expressed as % Cell Viability = O.D._ActD+test drugs_/O.D._ActD_. Effect of the GWE on TNF-α activity was evaluated using L929 cell bioassay in the presence of 1 μg/mL Actinomycin D (ActD) as described by Kiemer *et al.* [20]. In brief, L929 cells were seeded at a density of 1.5 × 10^4^ cells/well in 100 μL of culture media. Following incubation for 24 h, the medium in the wells was replaced with fresh medium containing ActD (2 μg/mL). After 1 h of preincubation with ActD, serial dilutions of test drugs and a final concentration of 0.2 ng/mL TNF-α was added. The plates were then incubated for an additional 24 h at 37°C and then MTT test was performed as described above. The TNF-α inhibitory effects of the test drugs were expressed as % inhibition = (O.D._ActD+TNF-α+test drugs_ – O.D._ActD+TNF-α_)/(O.D._ActD_ – O.D._ActD+TNF-α_).

### *In vivo* anti-inflammatory activity of GWE

Anti-inflammatory activities of GWE were tested using rat carrageenan-induced hind paw edema and mouse xylene-induced ear edema experimental models. The animal experiments were carried out at Yunnan Institute of Materia Medica (YIMM) and approved by the Animal Experimental Ethics Committee of YIMM (Approval Number: SCXK (Yunnan) 2005–0008). Based on the recommended dose of the raw drug for human (9 g/d, China Pharmacopoeia Committee, 2010) [1], the drug dose used for the animals was calculated to be 6.75 g/kg/d. The animals were kept in controlled environments and maintained on standard pellet diet and water *ad libitum*.

Carrageenan-induced rat hind paw edema model: Male Sprague–Dawley (SD) rats, weighting 160–210 g, were used. For each experiment, 30 SD rats were randomized into 3 groups, untreated control (water), treatments with GWE (1.69 g/kg/d) and reference drug control aspirin (0.1 mg/kg/d). Test drugs were administrated intragastrically each day for 5 consecutive days. One hour after the last drug dosing, each rat was injected with 0.1 mL freshly prepared suspension of 1% carrageenan in physiological saline into subplantar tissue of the right hind paw. Paw edema was then measured at 1, 2, 4, and 6 h after induction of inflammation.

Xylene-induced mouse ear edema model: Male ICR mice weighting 18–20 g were used. For each experiment, 30 ICR mice were randomized into 3 groups, untreated control (water), treatments with GWE (1.69 g/kg/d) and reference drug control aspirin (0.1 mg/kg/d). Test drugs were administrated intragastrically each day for 5 consecutive days. One hour after the last drug dosing, 50 μL xylene was applied to both anterior and posterior surfaces of the right ear for another hour. Subsequently, discs of 8 mm diameter were removed from each ear and weighed in a balance. The swelling was estimated as the difference in weight between the punches from right and left ears.

### Statistical analysis

The *In vivo* data are represented as Mean ± SE (10 animals per group). The *In vitro* assay are comparison between groups was made by One-way ANOVA, The differences with p < 0.05 or p < 0.01 were considered significant or highly significant, respectively.

## Results

**Figure 2:**
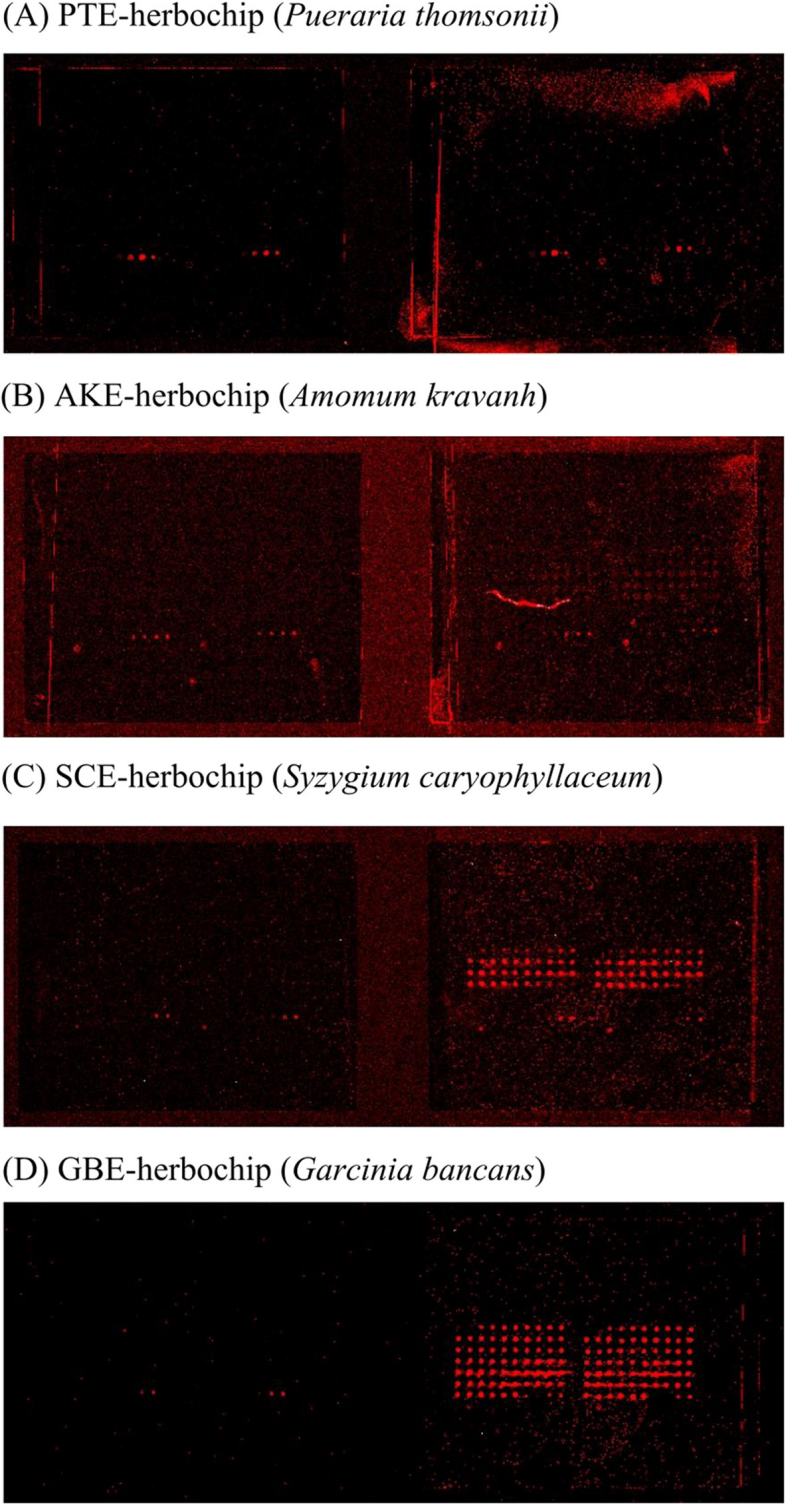
Binding signals of representative herbochips probed by β-TNF-α. The images were visualized by Cy5-labeled streptavidin after the binding of β-TNF-α to **(A)** PTE-, **(B)** AKE-, **(C)** SCE-, and **(D)** GBE-herbochips, which were fabricated with extracts from *Pueraria thromsonii*, *Amumon kravanh*, *Syzygium caryophyllaceum*, and *Garcinia bancans*, respectively. These herbochips chosen to represent different binding signal strengths from negative (−), weak (+), moderate (++), and strong (+++); as marked in Table 1.

**Figure 3:**
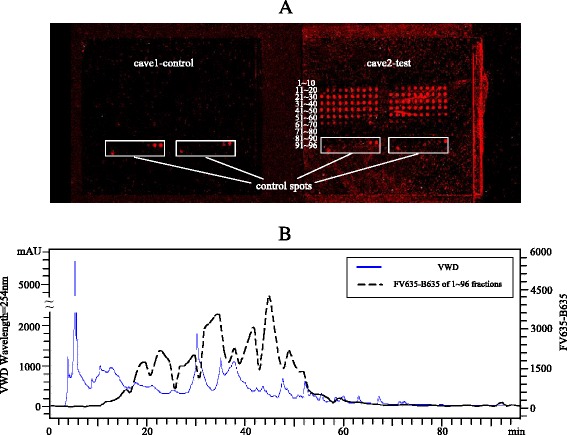
Binding signals of GWE-herbochip probed by β-TNF-α. **(A)** The image was visualized by Cy5-labeled streptavidin after the binding of β-TNF-α to the GWE-herbochip as described in Methods. **(B)** The fluorescence intensity of the image was quantized by a scanner at 635 nm presented (───) together with the original HPLC profile monitored at 254 nm (− − −−). The controls spots in **(A)** are 4, 10, 50, 250 ng/mL biotin in Optifix I, 1 μg/mL SA-Cy5 in Optifix II (positive control) and Optifix I (negative control) were spotted onto the slide in the same format shown in Figure 1. Positive signals indicated binding activity of the fractions to TNF-α.

Herein, a refined Herbochip® screening platform was constructed and reported. Treatment of the polystyrene plastic slides with glutaraldehyde led to formation of free aldehyde groups on the surface which subsequently reacted with 1,4-butanediol diglycidyl ether. The resulted terminally located epoxyl groups would then be able to react with carboxyl-, hydroxyl-, amino-, and sulfhydryl-groups to form covalent bonds. Thus most biological molecules including nucleic acids, proteins, and small molecules containing aforementioned functional groups would be conjugated onto the surface-activated slides (blank chips) when presented. The formatting of the HPLC fractions onto the chip was presented in Figure 1. After arraying, the unreacted epoxyl groups were blocked by ethanolamine in order to suppress background staining. TNF-α binding activity of the HPLC fractionated herbal extracts was unveiled by probing the herbochips with biotinylated TNF-α, which was then visualized by Cy5-conjugated streptavidin. Hybridization controls were provided by the control spots on each chips (Figure 1, C1-C6). C1-C4 samples containing different concentrations of biotinamidocaproyl hydrazide were used to indicate proper binding between biotin and Cy5-conjugated streptavidin. While C5 and C6 samples were used as internal negative and positive control, respectively (Figure 1). It is also worth to note that Cave 1 on each chip was left blank (no target protein was added) to serve as an additional control for the hybridization step (Figures 2 and 3, left panels).

A total of 82 selected herbs were made into herbochips and 46 herbs were found to possess TNF-α binding activities (Table 1). As shown in Figure 2, the signals of four herbochips, namely PTE (*Pueraria thromsonii* extract)-, AKE (*Amomum kravanh* extract)-, SCE (*Syzygium caryophyllaceum* extract)- and GBE (*Garcinia bancans* extract)-herbochips, were chosen to represent different binding signal strengths from negative binding (−) to strong binding (+++). Likewise, TNF-α binding activity of GWE-herbochip was shown in Figure 3. The red spots, corresponding to fractions 12 to 68, indicated TNF-α binding (Figure 3A). Since the binding activity was detected in a broad range of fractions and only very little materials were eluted after fraction 68, the GWE, with the typical HPLC profile shown in Figure 3B, was used in all following experiments without further fractionation.

**Figure 4:**
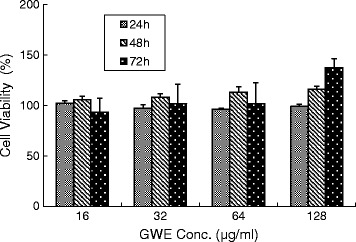
Cell viability at different concentrations of GWE. L929 cells were treated with indicated concentrations of GWE for up to 72 h as described in Methods. After treatments, cell viability was determined by MTT assays. The values show are mean ± S.E.M. from three independent experiments, no significance was found between groups.

**Figure 5:**
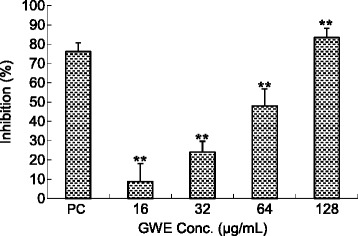
TNF-α inhibitory effects of the GWE. L929 cells were treated with indicated concentrations of GWE in the presences of 1 ng/mg TNF-α. After 24 h of treatment, cell viability was determined by MTT assays and TNF-α inhibitory effects of the test drugs were expressed as % inhibition = (O.D._ActD+TNF-α+test drugs_ – O.D._ActD+TNF-α_)/(O.D._ActD_ – O.D._ActD+TNF-α_). PC: Positive Control, 5 μg anti-TNF-α antibody. The values show are mean ± S.E.M. ($n$ =6). **$P$< 0.001 compared with the normal control group.

The cytotoxic effects of GWE on L929 cells were assayed in cultures exposed to GWE at indicated concentrations for 24, 48, and 72 h. As shown in Figure 4, the test drug GWE was found to be non-toxic at doses up to 128 μg/mL, at which slight enhancement of cell proliferation was found instead. In a slightly modified experimental protocol, the TNF-α sensitive L929 cells were also used for the assessment of the anti-TNF-α activity of GWE. It was found that GWE exhibited anti-TNF-α activity in a dose dependent manner, the degree of inhibition increased from 8.75% to 93.32% when the drug doses increased from 16 to 128 μg/mL (Figure 5).

In the carrageenan-induced hind paw edema assay, GWE inhibited swelling significantly 1 h post carrageenan injection (Table 2). In the control rats, the average swelling (n = 10) was 0.10 mL at 1 h, rose to 0.51 mL at 2 h, peaked at 0.66 mL at 4 h, and subsided to 0.40 mL at 6 h post-carrageenan. In the GWE-treated samples, the swelling rose from 0.07 mL to 0.14 mL after 2 h of induction and subsided rapidly to the baseline thereafter. The degrees of inhibition were calculated to be 30%, 73%, 85%, and 80% at 1, 2, 4, and 6 h post-carrageenan, respectively. This anti-inflammatory effect of GWE, at a dose of 1.69 g/kg/d, which was equivalent to 45 times of recommended dose for human (9 g raw drug/d), was found to be stronger than the positive control aspirin used at 0.10 mg/kg/d (Table 2). In fact, the anti-inflammatory effect of aspirin was only moderate and disappeared at 6 h post-carrageenan. These data indicated that when the animals were pre-fed with GWE, the carrageenan-induced hind paw edema could be significantly suppressed. The effect of GWE on xylene-induced mouse ear edema was shown in Table 3. Pretreatment of mice with GWE and aspirin resulted in a statistically significant reduction of xylene-induced edema. The degree of inhibition was calculated and it was found that 1.69 g/kg/d of GWE reduced swelling by 33.3% (p < 0.01, compared to the control). The positive control, aspirin (0.1 mg/kg/d), showed a 35.6% reduction in swelling (p < 0.01) (Table 3).

**Table 2:** **Effect of GWE on carrageenan-induced hind paw edema in rats**

**Test sample**	**Dose (kg/d)**	**Degree of swelling (mL)**			
		**1 h**	**2 h**	**4 h**	**6 h**
Control	−	0.10 ± 0.11	0.51 ± 0.07	0.66 ± 0.10	0.40 ± 0.06
GWE	1.69 g	0.07 ± 0.06	0.14** ± 0.08	0.10** ± 0.06	0.08** ± 0.06
Aspirin	0.10 mg	0.11 ± 0.12	0.39* ± 0.12	0.49* ± 0.14	0.39 ± 0.11

Data represent mean ± S.E.M. (n = 10). *p < 0.05; **p < 0.01.

**Table 3:** **Effect of GWE on xylene-induced ear edema in mice**

**Test sample**	**Dose (kg/d)**	**Degree of swelling (mL)**	**Inhibition (%)**
Control	−	8.7 ± 2.7	−
GWE	1.69 g	5.8 ± 2.2	33.3**
Aspirin	0.10 mg	5.6 ± 2.2	35.6**

Data represent mean ± S.E.M. (n = 10). *p < 0.05; **p < 0.01.

## Discussion

We have reported a microarray-based drug screening platform referred to as the Herbochip® for target-based discovery of natural compounds for functional characterization and therapeutic uses. The application of this platform was validated using TNF-α as the target for screening and one of the results, namely the anti-inflammatory fractions of the *G. wilfordii* extract (GWE), was further explored. The Herbochip® technology reported here was basically a reverse screening method. Unlike most high-throughput-screening (HTS) formats, in which large chemical libraries were screened against biological targets *via* the use of automation, miniaturized assays and large-scale data analysis [21,22], the Herbochip® platform employed a reverse screening format, in which the potential leads in herbal extract(s) were immobilized and selected protein drug target was used as the probe for the screening process [23]. In this Herbochip® platform the positive controls were spots that contained different concentrations of biotin (Figure 1, C1-C4, and C6). Additionally, one of spots in each cave (Figure 5, C5, where no biotin was present) and one of the caves (Cave 1) on each chip (in which no biotinylated target protein was added) served as negative controls. Together with the blocking step, herbochips constructed this way validated specific bindings between the immobilized ligands and the protein targets.

During the course of this study, thousands of different herbochips including the GWE-herbochip reported in details here, were fabricated. The protein drug target TNF-α was employed to screen 82 herbochips and the hit rate was as high as 56% (46/82) when herbs reported to have anti-inflammatory activity were counted. As mentioned, the platform was initially used to screen for herbal species that inhibit CYP450 3A4, an important enzyme for the metabolism of a broad range of therapeutic agents [18]. In addition to CYP450 3A4 and TNF-α, preliminary positives were obtained for other protein targets including vessel epithelial growth factor (VEGF), glucose-regulated protein78 (GRP78), receptor activator of NF-κB ligand (RANKL), *etc*., indicating that this novel HTS platform could be applied to a wide range of known drug targets. It is well known that the disease target of most, if not all, of herbal medicines remain unknown, the utilization of this target-based drug screening platform on developments of herbal medicine is definitely worth further explored.

TNF-α plays a central role in most inflammatory responses and it is a validated target for drugs against a number of chronic inflammatory disorders resulting from immune dysregulations such as Crohn’s disease, ulcerative colitis, ankylosing spondylitis, rheumatoid arthritis (RA), [7,24,25]. Using TNF-α as a probe together with subsequent assays in cell and animal models, TNF-α inhibitory activity and potential anti-RA activity in GWE were thus identified and correlated. We have tried to fractionate the GWE by solvent partition techniques and found that most of the anti-RA activity was partitioned into an acetyl acetate fraction. Unfortunately, the activity greatly diminished in the fraction therefore only the crude GWE was used for the studies reported. It is thus recommended that the total ethanolic extract of GW is to be used for further anti-RA drug development. Judging from the original chip profile shown in Figure 2, the TNF-α binding/inhibitory activity encompassed a large number of HPLC fractions, it is conceivable that the active ingredients consisted of a large derivatives may be a few natural compounds with similar structures. The actual therapeutic effects shown in the animal studies might result from the synergistically effects of this mixture, thus explained the diminishment of TNF-α inhibitory activity whenever the components were separated by any form of fractionation. It is worth to note that such phenomenon is not uncommon during the development of herbal medicines by Western approaches. In summary, these studies lead us to conclude that the anti-rheumatoid activity of GWE can be attributed to its inhibitory effect on the key inflammatory factor, TNF-α. Our results contribute towards validation of the traditional use of GWE in the treatment of RA and other inflammatory joint disorders, which in turn may facilitate further application traditional Chinese medicines in target-based therapies.

## Conclusion

We have thus validated effectiveness of the Herbochip® drug screening platform using TNF-α as a molecular target. Subsequent experiments on GWE lead us to conclude that the anti-RA activity of GWE can be attributed to inhibitory effect of GWE on the key inflammatory factor, TNF-α. Our results contribute towards validation of the traditional use of GWE in the treatment of RA and other inflammatory joint disorders.
